# The rare *C9* P167S risk variant for age-related macular degeneration increases polymerization of the terminal component of the complement cascade

**DOI:** 10.1093/hmg/ddab086

**Published:** 2021-03-30

**Authors:** O McMahon, T M Hallam, S Patel, C L Harris, A Menny, W M Zelek, R Widjajahakim, A Java, T E Cox, N Tzoumas, D H W Steel, V G Shuttleworth, K Smith-Jackson, V Brocklebank, H Griffiths, A J Cree, J P Atkinson, A J Lotery, D Bubeck, B P Morgan, K J Marchbank, J M Seddon, D Kavanagh

**Affiliations:** Complement Therapeutics Research Group, Translational and Clinical Research Institute, Newcastle University, Newcastle upon Tyne, NE2 4HH, UK; National Renal Complement Therapeutics Centre, Royal Victoria Infirmary, Newcastle upon Tyne, NE1 4LP, UK; Complement Therapeutics Research Group, Translational and Clinical Research Institute, Newcastle University, Newcastle upon Tyne, NE2 4HH, UK; National Renal Complement Therapeutics Centre, Royal Victoria Infirmary, Newcastle upon Tyne, NE1 4LP, UK; Department of Ophthalmology and Visual Sciences, University of Massachusetts Medical School, Worcester, MA 01655, USA; Complement Therapeutics Research Group, Translational and Clinical Research Institute, Newcastle University, Newcastle upon Tyne, NE2 4HH, UK; National Renal Complement Therapeutics Centre, Royal Victoria Infirmary, Newcastle upon Tyne, NE1 4LP, UK; Department of Life Sciences, Sir Ernst Chain Building, Imperial College London, London SW7 2AZ, UK; Division of Infection and Immunity, School of Medicine, Systems Immunity Research Institute, Cardiff University, Heath Park, Cardiff CF14 4XN, UK; Department of Ophthalmology and Visual Sciences, University of Massachusetts Medical School, Worcester, MA 01655, USA; Divisions of Nephrology and Rheumatology, Department of Medicine, Washington University, St Louis, MO 63110, USA; Complement Therapeutics Research Group, Translational and Clinical Research Institute, Newcastle University, Newcastle upon Tyne, NE2 4HH, UK; National Renal Complement Therapeutics Centre, Royal Victoria Infirmary, Newcastle upon Tyne, NE1 4LP, UK; Biosciences Institute, Newcastle University, Newcastle upon Tyne, NE1 3BZ, UK; Biosciences Institute, Newcastle University, Newcastle upon Tyne, NE1 3BZ, UK; Complement Therapeutics Research Group, Translational and Clinical Research Institute, Newcastle University, Newcastle upon Tyne, NE2 4HH, UK; National Renal Complement Therapeutics Centre, Royal Victoria Infirmary, Newcastle upon Tyne, NE1 4LP, UK; Complement Therapeutics Research Group, Translational and Clinical Research Institute, Newcastle University, Newcastle upon Tyne, NE2 4HH, UK; National Renal Complement Therapeutics Centre, Royal Victoria Infirmary, Newcastle upon Tyne, NE1 4LP, UK; Complement Therapeutics Research Group, Translational and Clinical Research Institute, Newcastle University, Newcastle upon Tyne, NE2 4HH, UK; National Renal Complement Therapeutics Centre, Royal Victoria Infirmary, Newcastle upon Tyne, NE1 4LP, UK; Clinical and Experimental Sciences, Faculty of Medicine, University of Southampton, Southampton SO16 6YD, UK; Clinical and Experimental Sciences, Faculty of Medicine, University of Southampton, Southampton SO16 6YD, UK; Divisions of Nephrology and Rheumatology, Department of Medicine, Washington University, St Louis, MO 63110, USA; Clinical and Experimental Sciences, Faculty of Medicine, University of Southampton, Southampton SO16 6YD, UK; Department of Life Sciences, Sir Ernst Chain Building, Imperial College London, London SW7 2AZ, UK; Division of Infection and Immunity, School of Medicine, Systems Immunity Research Institute, Cardiff University, Heath Park, Cardiff CF14 4XN, UK; Complement Therapeutics Research Group, Translational and Clinical Research Institute, Newcastle University, Newcastle upon Tyne, NE2 4HH, UK; National Renal Complement Therapeutics Centre, Royal Victoria Infirmary, Newcastle upon Tyne, NE1 4LP, UK; Department of Ophthalmology and Visual Sciences, University of Massachusetts Medical School, Worcester, MA 01655, USA; Complement Therapeutics Research Group, Translational and Clinical Research Institute, Newcastle University, Newcastle upon Tyne, NE2 4HH, UK; National Renal Complement Therapeutics Centre, Royal Victoria Infirmary, Newcastle upon Tyne, NE1 4LP, UK

## Abstract

Age-related macular degeneration (AMD) is a complex neurodegenerative eye disease with behavioral and genetic etiology and is the leading cause of irreversible vision loss among elderly Caucasians. Functionally significant genetic variants in the alternative pathway of complement have been strongly linked to disease. More recently, a rare variant in the terminal pathway of complement has been associated with increased risk, Complement component 9 (*C9*) P167S. To assess the functional consequence of this variant, C9 levels were measured in two independent cohorts of AMD patients. In both cohorts, it was demonstrated that the P167S variant was associated with low C9 plasma levels. Further analysis showed that patients with advanced AMD had elevated sC5b-9 compared to those with non-advanced AMD, although this was not associated with the P167S polymorphism. Electron microscopy of membrane attack complexes (MACs) generated using recombinantly produced wild type or P167S *C9* demonstrated identical MAC ring structures. In functional assays, the P167S variant displayed a higher propensity to polymerize and a small increase in its ability to induce hemolysis of sheep erythrocytes when added to C9-depleted serum. The demonstration that this *C9* P167S AMD risk polymorphism displays increased polymerization and functional activity provides a rationale for the gene therapy trials of sCD59 to inhibit the terminal pathway of complement in AMD that are underway.

## Introduction

Age-related macular degeneration (AMD) is a complex neurodegenerative disease with both behavioral and genetic underpinnings and is the leading cause of irreversible vision loss in elderly Caucasian populations (1). It impairs quality of life and daily activities, and the burden of visual loss due to this disease is expected to increase by almost 50% in the next 20 years as the population of individuals over the age of 60 expands (2,3). Common and rare variants in more than 30 genetic loci have been associated with the disease (4–7) with the complement system being one of the key implicated pathways.

The complement system is an ancient innate immune response system with three main activation pathways: the classical, mannose-binding lectin and alternative pathways (AP), and a common terminal pathway. Genetic studies have predominantly identified components (*C3*, *CFB*) and regulators (*CFH*, *CFI*) of the AP as being involved in the development and progression of AMD (4,5,8–20). The predominance of risk association with the AP reflects its role in complement amplification, accounting for 80–90% of terminal pathway activation regardless of the activating pathway (21).

The deposition of the terminal pathway complex in drusen, the choriocapillaris and the retinal pigment epithelium has long been described in AMD (22,23). More recently, genetic components of the terminal pathway of complement have been associated with AMD. A genetic association between C9 and AMD was first described in the Japanese population. In Japan, complete C9 deficiency is one of the most frequent genetic disorders, with an incidence of approximately one homozygote in 1000 (24), and is associated with increased risk of meningococcal sepsis (25). A specific mutation, R95X, is responsible for the majority of Japanese C9 deficiency with 6.7% of the population carrying this mutation (26). Nishiguchi *et al.* (27) found that haploinsufficiency of C9 caused by this mutation was associated with reduced risk of neovascular AMD (odds ratio = 0.2) in a single cohort.

The first risk C9 polymorphism (P167S) associated with advanced AMD (AAMD) was described by Seddon *et al*. in a targeted sequencing study of 681 genes in 2493 individuals with independent replication in 5115 samples. Carriers of this mutation had a 2.2-fold increased risk of developing AMD (5). This association has been confirmed in subsequent studies (4,28). The risk variant *C9* P167S also tended to increase the risk of progression from early and intermediate levels of AMD to AAMD in prospective studies (29–31), although it was not independently related after adjusting for all other behavioral and genetic factors. Additional rare variants in *C9* were later described in AMD (32,33).

Recent work has suggested that the AMD *C9* risk polymorphism P167S is associated with increased circulating levels of the protein (32,33). Conversely, in two independent cohorts, this study demonstrated decreased circulating C9 levels, but increased functional activity of the risk variant.

## Results

### Measurement of C9 plasma levels

The plasma level of C9 was measured in 113 individuals carrying the P167S variant as heterozygotes and 114 individuals homozygous for the wild-type sequence from a North American cohort. Samples were age and AMD status matched (P167S heterozygous: 40 non-AAMD; 73 AAMD/wild type: 41 non-AAMD; 73 AAMD).

**Figure 1:**
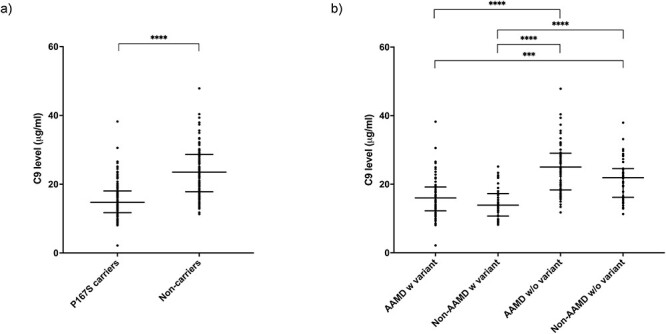
Plasma C9 levels in the North American cohort. (a) Plasma levels by P167S status irrespective of phenotype. The median C9 plasma levels were as follows: P167S, 14.7 μg/ml; no variant, 23.5 μg/ml; (b) C9 plasma levels by P167S variant and AAMD status. The median C9 plasma levels were as follows: AAMD with P167S, 16 μg/ml; non-AAMD with P167S, 13.9 μg/ml; AAMD without variant, 25 μg/ml and non-AAMD without variant, 21.9 μg/ml. Statistics shown include comparison of the median by a Mann–Whitney test (a) and Dunn’s multiple comparisons test (b). Interquartile range and median are shown by bars. Statistically significant results are indicated by (***) or (****). Defined as ^****^P < 0.0001, ^***^P < 0.001.

When C9 plasma levels were analyzed based on P167S genotype irrespective of AAMD status, the median plasma C9 concentration was lower in those carrying the variant (14.7 μg/ml) compared to those with wild-type sequence (23.5 μg/ml) (*P* < 0.0001) (Fig. 1a). This significantly lower level in those with the P167S variant was seen in individuals with AAMD (16 μg/ml) and without AAMD (13.9 μg/ml) compared to those without the variant with AAMD (25 μg/ml) and without AAMD (21.9 μg/ml) (Fig. 1b). There was no significant difference between C9 levels in participants with AAMD and the *C9* P167S variant compared to those in the non-AAMD group with the variant. Likewise, no significant difference was found between those with AAMD without the C9 P167S variant and those in the non-AAMD group without the variant (Fig. 1b).

Previously Kremlitzka *et al*. (32,33) had suggested the P167S variant resulted in increased serum levels. We initially hypothesized that this discrepancy between the studies may reflect the specificity of the capture monoclonal antibody (mAb), B7, used in the enzyme-linked immunosorbent assay (ELISA). Substituting the B7 mAb for an in-house generated anti-C9 Ab (10E10) allowed a repeat analysis to be undertaken in an age-matched subgroup of our initial population and again this demonstrated a significantly lower C9 level in those individuals with the P167S variant (Supplementary Material, Fig. S1).

To ensure that the B7 and 10E10 mAb did not bind to the same epitope, a competition assay between these two antibodies was undertaken revealing a slight loss of signal suggesting overlapping epitope targets (Supplementary Material, Fig. S2).

Thus, a third ELISA was performed using the same capture mAb and detection polyclonal Ab as in the Kremlitzka manuscript with similar conditions (32). Again, it was demonstrated that that those individuals with the P167S variant had lower levels compared to those without (Supplementary Material, Fig. S3). Competition assays were undertaken, which demonstrated that B7 and the X197 anti-C9 mAb used in this latter study did not compete for the same binding epitope (Supplementary Material, Fig. S2).

For additional verisimilitude, semi-quantitative densitometry of western blots using polyclonal Abs to probe C9 was performed in a subset of patients to remove any confounding effect of the mAb binding site. The results correlated with the ELISA data, demonstrating reduced C9 levels in the presence of the P167S variant (Supplementary Material, Fig. S4).

**Figure 2:**
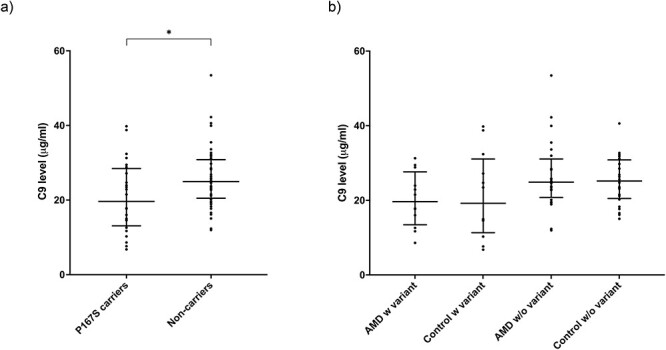
Plasma C9 levels in the UK cohort. (a) Plasma levels by P167S status irrespective of phenotype. The median C9 plasma levels were as follows: P167S, 19.7 μg/ml; no variant, 24.9 μg/ml; (b) C9 plasma levels by P167S variant and AAMD status. The median C9 plasma levels were as follows: AMD with P167S, 19.7 μg/ml; Control with P167S, 19.2 μg/ml; AMD without variant, 24.9 μg/ml; and control without variant, 25.2 μg/ml. Statistics shown include comparison of the median by a Mann–Whitney test (a) and Dunn’s multiple comparisons test (b). Interquartile range and median are shown by bars. Statistically significant results are indicated by (*). Defined as ^*^P < 0.05.

Plasma levels of C9 were then analyzed in an independent validation cohort from the United Kingdom (P167S heterozygous: 12 control, 12 AMD; wild type: 24 control, 24 AMD). Again, the median C9 plasma concentration in those heterozygous for the P167S variant (19.7 μg/ml) was significantly lower than those homozygous for WT *C9* (24.9 μg/ml) (*P* = 0.0188) (Fig. 2a). When subclassified by phenotype, lower C9 levels in those with the P167S variant were seen in individuals with AMD (19.7 μg/ml) or without AMD (19.2 μg/ml) compared to those without the variant with AMD (24.9 μg/ml) and without AMD (25.2 μg/ml) (Fig. 2b), although with the smaller cohort size this did not reach statistical significance.

### Measurement of sC5b-9 plasma levels

**Figure 3:**
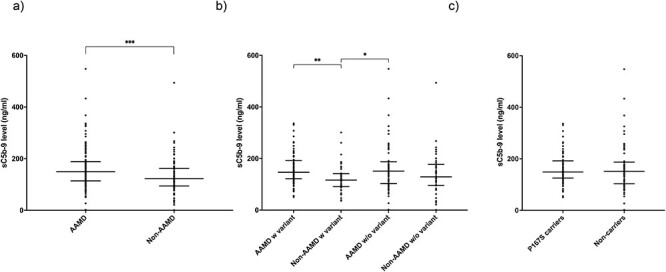
Plasma sC5b-9 levels from North American cohort. (a) sC5b-9 plasma levels by AAMD status irrespective of genotype. The median C9 plasma levels were as follows: AAMD, 149.3 ng/ml; non-AAMD, 122.4 ng/ml. (b) sC5b-9 plasma by P167S variant and AAMD status. The median sC5b-9 plasma levels were as follows: AAMD with P167S, 146.7 ng/ml; non-AAMD with P167S, 116.5 ng/ml; AAMD without variant, 151.4 ng/ml; and non-AAMD without variant, 129.1 ng/ml. (c) Plasma sC5b-9 levels by P167S status irrespective of phenotype. The median sC5b-9 plasma levels were as follows: P167S, 149 ng/ml; no variant, 151.4 ng/ml. Statistics shown include comparison of the median by a Mann–Whitney test (a, c) and Dunn’s multiple comparisons test (b). Median with interquartile range is shown by bars. Statistically significant results are indicated by (*), (**) or ***). Defined as ^*^P < 0.05, ^**^P < 0.01 and ^***^P < 0.001.

The plasma level of sC5b-9 was measured in all 227 individuals in the North American cohort, and there was a significantly higher median sC5b-9 level in patients with AAMD regardless of P167S genotype (AAMD: 149.3 ng/ml, non-AAMD: 122.4 ng/ml, *P* = 0.0004) (Fig. 3a). When analyzed by genotype and phenotype, the median sC5b-9 level was significantly higher in the AAMD group with the P167S variant compared to the non-AAMD group with the variant (146.7 and 116.5 ng/ml, respectively; *P* = 0.0058). Further, there was a significantly higher median level in those with AAMD without the variant compared to the non-AAMD group with the variant (151.4 and 116.5 ng/ml, respectively; *P* = 0.012) (Fig. 3b). When analyzed only by P167S genotype, there was no significant difference in the median sC5b-9 level between P167S variant carriers (149 ng/ml) and non-carriers (151.4 ng/ml) (Fig. 3c). There was a weak but significant correlation (*P* = 0.0079; *r* = 0.175) between sC5b-9 levels and C9 levels (Supplementary Material, Fig. S5).

### Measurement of C9 from transiently transfected CHO cell culture supernatant

**Figure 4:**
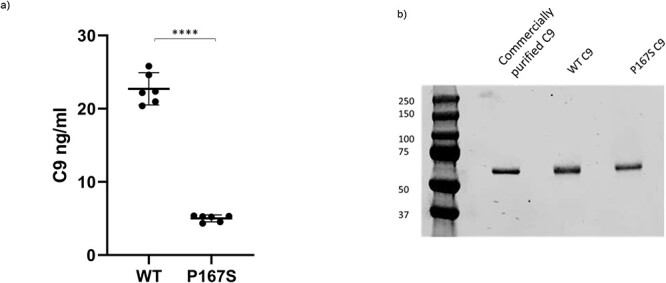
Expression and purification of recombinant C9 proteins. (a) Multiple independent transient transfections of CHO cell lines with the pDR2ΔEF1α vector containing WT (*n* = 6) or P167S variant (*n* = 6) C9 were performed. The C9 level in cell supernatants was measured, and there was a significantly lower C9 level of the P167S compared to WT (5 vs. 22.7 ng/ml, *P* < 0.0001). Mean with 95% confidence interval is shown by bars. (b) Stable transfections of recombinant WT and P167S CHO lines were produced, and C9 was purified by affinity chromatography and size exclusion prior to non-reduced SDS PAGE and Coomassie staining to assess purity. ^****^P < 0.0001.

C9 levels in supernatants from multiple independent transiently transfected CHO cell cultures were measured. CHO cells transfected with the WT vector produced significantly more C9 compared to those transfected with the P167S variant (mean: 22.7 vs. 5.0 ng/ml; *P* < 0.0001) (Fig. 4a). For functional experiments, stably transfected cell lines were used and the recombinant protein was purified to homogeneity (Fig. 4b) and identity confirmed by mass spectrometry.

### Functional assessment of variant

**Figure 5:**
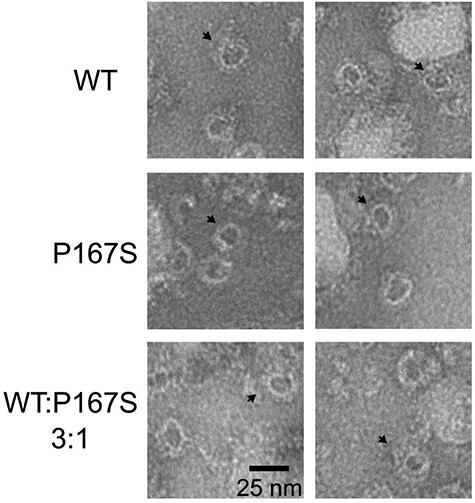
Negative staining EM of WT, P167S and 3:1 WT:P167S mix membrane attack complex formation. Representative negative stain images of MAC containing either WT, P167S or a 3:1 WT:P167S ratio mix of C9 assembled on DOPC:DOPE monolayers. Examples of oligomeric MAC complexes are indicated on each image (black arrow). Scale bar, 25 nm.

To determine the ability of the P167S variant to form MAC, we incubated recombinant WT or P167S C9 with C5b-8 complexes pre-assembled on lipid monolayers. Complexes were negatively stained and imaged by electron microscopy (EM). These data show that P167S C9 associates with membrane-bound C5b-8 complexes and polymerizes to form oligomeric structures similar to wild-type MAC (Fig. 5).

**Figure 6:**
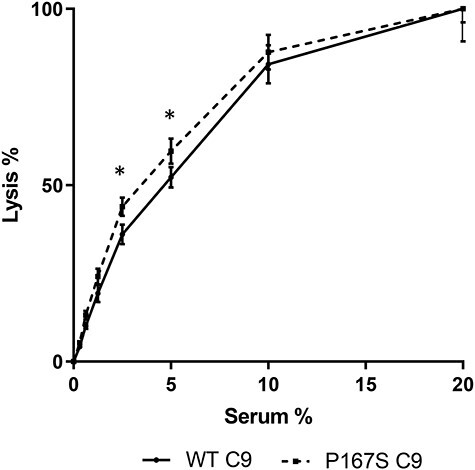
Hemolytic activity of recombinant C9 proteins. Sensitized SRBCs were incubated with C9-depleted serum reconstituted with recombinant C9 WT or P167S. Multiple independent assays were performed (*n* = 8) in triplicate and averaged. CH50 was 4.64% (or 2.62 μg/ml) for WT and 3.72% (or 2.05 μg/ml) for P167S. Statistically significant results are indicated by (*).

To assess the hemolytic activity of the variant, C9-depleted serum was reconstituted with equimolar concentrations of either recombinant WT or P167S C9, serially diluted and incubated with sheep red blood cells (SRBCs). The P167S C9 caused a slight increase in lysis of SRBC when compared with the WT C9 [*n* = 8 in triplicate, *P* < 0.05, CH50 was 4.64% (or 2.62 μg/ml) for WT and 3.72% (or 2.05 μg/ml) for P167S] (Fig. 6).

**Figure 7:**
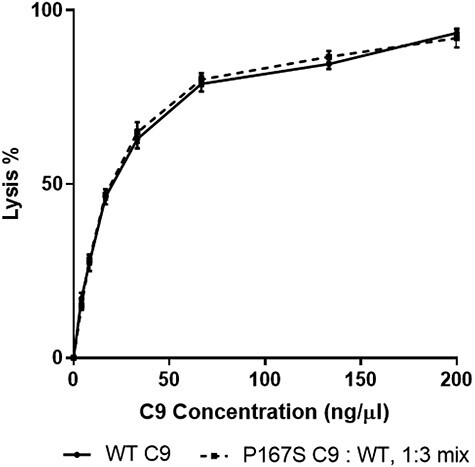
Lytic activity of WT and a 3:1 WT:P167S ratio. Guinea pig erythrocytes were incubated serially with C5b6, C7 and C8 followed by either WT C9 or a 3:1 ratio of WT/P167S. Multiple independent assays were performed (*n* = 5) in triplicate and averaged. No statistical significance was demonstrated.

With such small differences in the hemolytic activity of P167S, we sought to test the hypothesis that the variant may act as a nidus around which WT C9 may more easily polymerize. Negative staining EM demonstrated that a 3:1 mix of WT:P167S C9 formed similar ring structures to either component individually (Fig. 5). Reactive lysis experiments using the same 3:1 WT:P167S ratio demonstrated no significant differences in hemolytic activity (Fig. 7), providing no evidence that the P167S variant exerts its effect *in vivo* through a focal point for WT polymerization.

**Figure 8:**
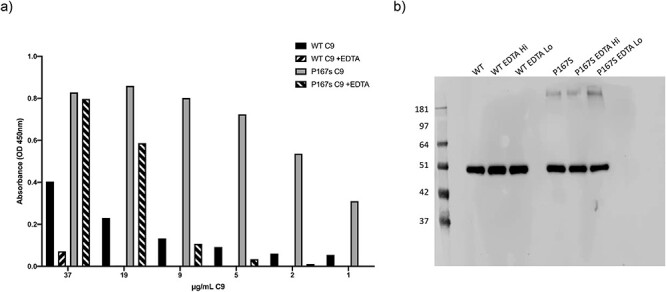
Polymerization of recombinant C9 proteins. (a) Freshly purified WT and P167S C9 were incubated at 37°C 1 h in the presence or absence of EDTA. The amount of polymerized C9 was quantified by in house ELISA. The P167S variant polymerized to a greater extent than WT protein and was more resistant to EDTA. (b) Confirmation of polymerization was also assessed with western blotting of freshly purified WT and P167S, incubated at 37°C for 1 h in the presence or absence of EDTA. High molecular weight species was only detected with the P167S variant.

The ability of recombinant WT and P167S variant C9 to polymerize was assessed in an in-house ELISA based on a C9 polymer neoepitope mAb. Purified recombinant C9 (WT and P167S) was subjected to size exclusion chromatography to remove any aggregates and to exchange buffer into assay diluent. On the same day, proteins were removed from ice and incubated at 37°C for 1 h in the presence or absence of EDTA. Polymers of C9 were detected by ELISA using an mAb that specifically detected neoepitope exposure in C9 polymers. WT C9 formed polymers at 37°C and this was inhibited by EDTA. The P167S variant polymerized more readily and inhibition by EDTA was only evident at low concentrations of C9 (Fig. 8a). A portion of the incubation was subjected to SDS PAGE, revealing the presence of polymers in the P167S sample, but not in the WT protein preparation (Fig. 8b).

**Figure 9:**
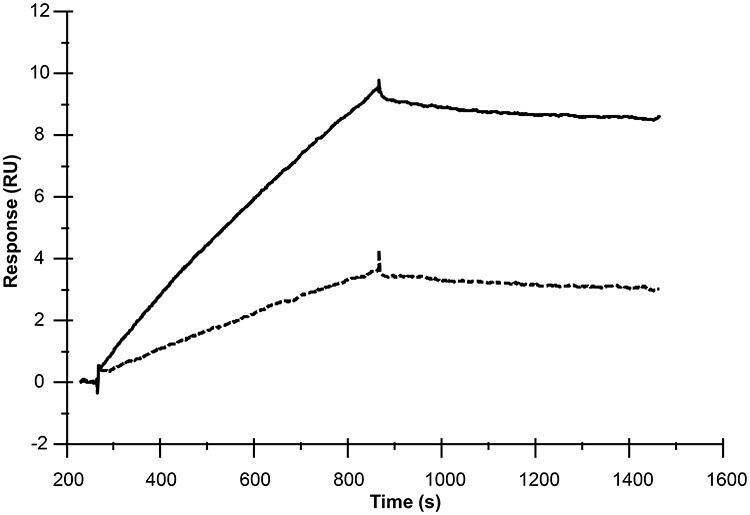
Surface plasmon resonance analysis of polymerization. Preparations of C9 were polished by size exchange chromatography to remove aggregates and flowed slowly (5 μl/min) at 37°C across a Biacore chip that had been immobilized with aE11 mAb specific for polymerized C9. Polymers of C9 formed by WT and P167S C9 were captured on the antibody. Solid line represents poly-C9 captured from the preparation of mutant P167S C9 and dashed line represents capture of poly-C9 in the WT preparation. The experiment was performed multiple times with different preparations of C9; a representative analysis is illustrated.

The aggregate-free preparations of WT and P167S C9 were also flowed across a Biacore chip on which the anti-neoepitope mAb had been immobilized. The flow was reduced to a very low rate (5 μl/min), and the temperature was raised to 37°C. When the variant C9 was flowed across the chip, more material was captured on the anti-neo mAb compared to WT, indicating higher levels of polymerization of P167S at 37°C (Fig. 9).

## Discussion

To assess the consequences of the complement *C9* rare genetic variant P167S in AMD, plasma C9 levels were initially measured in the US cohort, revealing a significantly lower median plasma C9 level associated with the P167S variant (14.7 vs. 23.5 μg/ml, *P* < 0.0001). This lower level in those with the P167S variant was seen in individuals with AAMD (16 μg/ml) or without AAMD (13.9 μg/ml), compared to those without the variant with AAMD (25 μg/ml) and without AAMD (21.9 μg/ml).

In contrast, a recently published study has suggested that the P167S risk variant was associated with increased circulating levels of C9. Geerlings *et al.* measured the serum C9 levels of ~90 individuals carrying the P167S rare genetic variant and demonstrated individuals with the P167S had higher average serum C9 levels (10.7 μg/ml) than controls with (6.6 μg/ml) or without (6.1 μg/ml) AMD.

The normal range of C9 in the literature is broad, ranging from 28 to 98 μg/ml (34–38). It has previously been demonstrated that C9 levels were 14% lower in females compared to males (39) and C9 levels have been reported to increase with age up to ~25% (39). The possibility of variations in levels due to age and sex differences prompted us to obtain a verification cohort from the UK (the Southampton cohort from the AMD Genomics Consortium database), and again in that replication cohort, those with the P167S variant had lower plasma concentrations compared to those without (19.7 vs. 24.9 μg/ml), irrespective of phenotype.

To assess if the decrease in circulating C9 levels was due to depletion, plasma sC5b-9 levels were analyzed in the US cohort. Increased systemic sC5b-9 has previously been reported in AMD (40), and this finding is replicated here. There was a significant increase in the median sC5b-9 level in those with AAMD versus those without AAMD (149.3 and 122.4 ng/ml, respectively; *P* = 0.0004); however, we did not see evidence of consumption associated with the variant with no significant difference found in sC5b-9 levels between P167S variant carriers and non-carriers.

That transiently transfected CHO cells produced significantly reduced P167S variant compared to cells transfected with a WT construct may suggest that the lower plasma C9 levels are due to reduced secretion *in vivo.* This is in keeping with the findings of Kremlitzka *et al.* who also demonstrated a non-significant trend to lower C9 secretions in HEK293 cells transfected with P167S compared to WT (32).

To test the functional activity of the P167S variant, hemolytic assays on sensitized sheep erythrocytes were undertaken. These demonstrated a very minor but significant increase in lytic ability of the variant C9 (Fig. 6). This is in contrast to the findings of Kremlitzka *et al.* (32) who reported reduced hemolytic activity from both recombinantly produced variant C9 and variant C9 from patients’ sera. A mixture of WT and variant C9 to reflect a more physiological situation did not see a significant increase in lysis, thus failing to provide any evidence that P167S acted as nidus for WT polymerization.

The P167S recombinant C9 spontaneously polymerized to a greater extent than the WT protein, and this polymerization was resistant to EDTA treatment. Whether this increased self-association observed is truly relevant to hierarchical MAC formation *in vivo* remains to be established although this increase polymerization in freshly gel filtered P167S did correlate with the increased lytic activity seen *in vitro*. Unsurprisingly, high molecular weight C9 species were not seen in plasma likely due to rapid clearance in the circulation (Supplementary Material, Fig. S6). These findings mirror those of Kremlitzka *et al.* (32) and prompted a structural analysis of the variant MAC formation. Despite these marked deferences in polymerization, there were no gross differences in MAC formation between WT and P167S C9 by negative staining EM (Fig. 5).

Although our study demonstrates that the P167S variant in *C9* is more active, it is associated with a decreased circulating plasma level. While this may be due to consumption of the protein, we did not find an increased sC5b-9 level associated with this genetic variant, similar to a previous report (32). In contrast, both our study and the previous report demonstrated decreased *in vitro* secretion of C9, suggesting an impact on synthetic/secretory pathways. In canonical terminal pathway formation, the limiting component of MAC hemolysis in serum is C5 (39,41); thus, the slight reduction in C9 level associated with this variant may not limit C5b-9 formation in this context.

**Figure 10:**
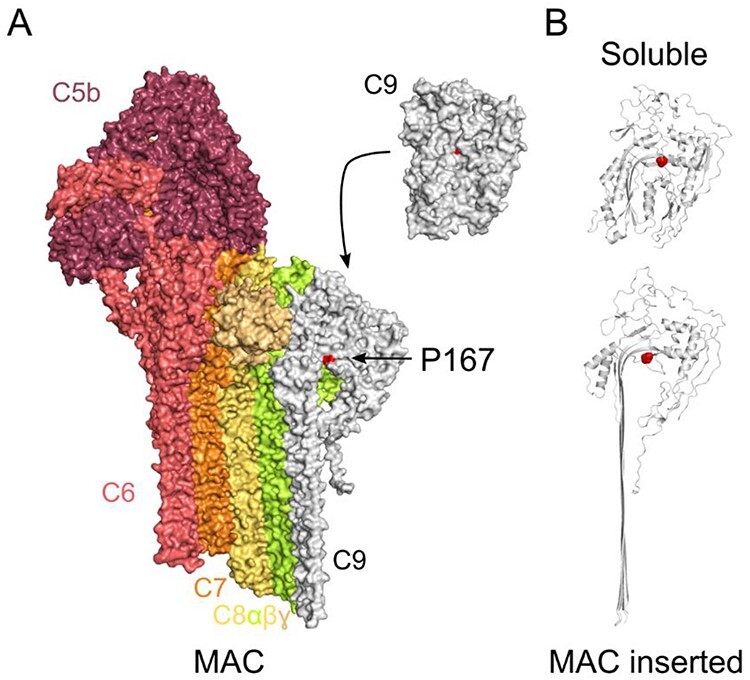
Localization of P167 on C9. (a) Structure of MAC and soluble C9. Only one of the 18 C9 in MAC is shown for clarity. All subunits are represented as surfaces and colored individually. The position of P167 (red) is highlighted on the surface of C9 (white). (b) Ribbon representation of soluble (top) and MAC inserted (bottom) C9. P167 side chain is shown as red spheres.

The impact of C9 polymerization as a consequence of the P167S variant in AMD is complex. The affected residue lies within the pore-forming membrane attack complex perforin (MACPF) domain of C9. Similar to other AMD-affected residues that influence C9 polymerization (32), P167 contributes to an interface between two adjacent C9 molecules in the MAC (Fig. 10) (42). In both the soluble (43) and membrane inserted forms of C9 (42), P167 forms part of a loop that latches across the central β-sheet of the MACPF domain. While the orientation of this loop is similar in both states (Fig. 10b), a proline mutation may also influence the geometry of this loop for an intermediate C9 conformation.

What is evident from our and Kremlitzka’s study (32) is that P167S variants have an increased propensity to oligomerize. While changing the propensity for C9 to oligomerize could result in less C9 available for MAC formation by promoting spontaneous self-polymerization of C9, our data show increased lytic activity of the P167S variant. A recent atomic force microscopy study investigating the kinetic drivers of MAC assembly shows that incorporation of the first C9 occurs on a much slower timescale than oligomer propagation (44). As CD59 blocks MAC formation by binding C8 and C9 of MAC precursor complexes, this difference in rate offers an opportunistic window for CD59 to act. Although the P167S C9 mutation is far from the CD59-binding site, it could impact the rate of unfurling C9 transmembrane hairpins, a requirement for polymerization (43) (Fig. 10). By speeding up the rate of the first C9, the window for CD59 to trap pore-formation narrows. The knock-on effects of this could explain the observed MAC-mediated cell damage in AMD.

C5b-9 deposition in drusen and the subretinal space was initially described by Anderson *et al*. (22). Further studies demonstrated that C5b-9 was heavily deposited on the choroidal neovascular membranes in wet AMD patients as well as the junction between the choriocapillaris and retinal pigment epithelium (RPE) of dry AMD patients (23). Increased C5b-9 deposition has been associated with the risk variant of the complement factor H (*CFH*) Y402H polymorphism both *in vivo* (45) and *in vitro* (46,47)*.* In individuals homozygous for the *CFH* 402H risk polymorphism, a ~70% greater deposition of C5b-9 on the retinal pigment epithelium/choroid relative to the low-risk individuals was reported (48). The demonstration of functionally significant risk and protective C9 alleles in AMD provides evidence supporting a causative role for C5b-9/MAC in AMD pathogenesis rather than as a bystander biomarker of upstream complement activation. In nucleated cells such as in the RPE, MAC deposition leads to a variety of cell signaling processes resulting in the secretion of cytokines and inflammatory molecules; this can ultimately cause the loss of the RPE, regression of the choriocapillaris and dry AMD (reviewed in (49)).

This study demonstrated that the C9 P167S risk variant for AMD had an increased polymerization propensity and slightly increased functional lytic activity. Although such *in vitro* studies cannot mimic completely the complexity of biological systems, together with other genetic studies highlighting C9 haploinsufficiency as protective for AMD and evidence of MAC deposits in areas of pathology, this study supports the importance of the terminal pathway of complement in AMD. Ultimately, the clinical studies currently underway of terminal pathway blockade using sCD59 with gene therapy (NCT03144999) may provide the definitive evidence for the role of MAC in AMD.

## Materials and Methods

### Patient sample collection and selection

The North American cohort included Caucasian participants in ongoing genetic and epidemiologic studies of macular degeneration who signed consent forms for the study approved by the institutional review boards (6,15,17,18,50–56). The presence or absence of AMD was determined based on clinical examination, fundus photography and optical coherence tomography. J.M.S. assigned grades for no AMD, early, intermediate and advanced stages of AMD using the Clinical Age-Related Maculopathy Grading System (50) as follows: Grade 1, no drusen or only a few small drusen; Grade 2, early maculopathy with small, hard drusen (2a) or retinal pigment alteration (2b) or both (2c); Grade 3, intermediate AMD with several intermediate drusen and/or presence of large, soft drusen; and AAMD with Grade 3b, drusenoid retinal pigment epithelial detachment; Grade 4, central or non-central geographic atrophy and Grade 5, neovascular AMD.

The UK AMD cohort included cases and controls who were examined and diagnosed by A.J.L. as previously described (16,57), before the cases were classified into the appropriate subcategories as per the AREDS system (58). Consent was obtained in accordance with the Declaration of Helsinki and was approved by South West Hampshire Local Research Ethics Committee (374/02/t and 150/03/t). Informed consent was obtained from all subjects, and all methods were carried out in accordance with the relevant guidelines and regulations of Research Ethics Committees.

### Genetic analysis

For the North American cohort, targeted sequencing of 681 genes including *C9* for samples from 1676 individuals with AAMD and 745 subjects without any signs of AMD, from a study cohort of over 5000 cases and controls, was previously undertaken (5). For the UK AMD case–control cohort, SNP-chip genotyping was performed as part of the sequencing of the AMD-EU-JHU cohort by the procedures outlined in the study by the AMD Gene Consortium (59). The Southampton AMD cohort contains 577 patients with AMD and 651 elderly controls.

### Plasma samples

EDTA plasma samples were obtained according to a standard protocol and stored at −140°C. In both cohorts, individuals were selected for the study on the basis of plasma availability.

### Enzyme-linked immunosorbent assay

#### C9 levels

Flat-bottomed 96-well plates (Maxisorp, ThermoFisher) were coated with 1 μg/ml mAb mouse anti-human C9 B7 (Prof B.P. Morgan, Cardiff University, Paisley, UK) or 10E10 (in house) in 100 mm bicarbonate/carbonate buffer pH 9.6 and incubated overnight at 4°C. The plates were then washed three times with PBST (0.1% tween in phosphate buffered saline) and blocked for 1 h with 200 μl of PBS/1% BSA/0.05% Na azide at room temperature (RT). The plate was washed again three times, and 100 μl of sample was added in duplicate and incubated for 1 h at RT. The standard was made up of seven 1:2 serial dilutions from 1 μg/ml purified C9 (CompTech, A126, Texas, USA). All samples including the standards were added in duplicate. The plate was washed again three times. 100 μl of polyclonal rabbit anti-human C9 (Abcam, ab71330, Cambridge, UK) diluted 0.5 μg/ml in block solution was added and incubated for 1 h at RT. The plate was washed again three times. 100 μl of HRP goat anti-rabbit IgG (Abcam, ab6721, Cambridge, UK) diluted 1/2000 in block solution was added and incubated for 1 h at RT. The plate was washed again three times before 100 μl of TMB substrate solution (Thermo Scientific, N301, Paisley, UK) was added and incubated for 10 min at RT. 100 μl of 10% H_2_SO_4_ was added to each well to stop the reaction. Plates were analyzed on a FLUOstar Optima (BMG Labtech, Aylesbury, UK) plate reader at 450 nm, and GraphPad Prism V8 was used to calculate concentrations by interpolation of a standard curve. Calculated intra-plate CV (%) and inter-plate CV (%) were 7 and 9, respectively.

#### SC5b-9 levels

A commercial kit was used according to manufacturer’s instructions (MicroVue: Quidel, San Diego, USA). Calculated intra-plate CV (%) and inter-plate CV (%) were 5 and 8, respectively.

#### Poly-C9 levels

Flat-bottomed 96-well plates (Maxisorp, ThermoFisher) were coated with 0.5 μg/ml human terminal complement complex-specific mAb aE11 (Hycult, HM2167, Uden, The Netherlands) in 200 mm bicarbonate/carbonate buffer pH 9.6 and incubated overnight at 4°C. The next day the solution was removed from the plate, and it was washed three times using PBS with 0.1% Tween 20. Two hundred μl of PBS/1% BSA/0.05% Na azide block solution was added to the plate and incubated for 1 h at RT. The plate was washed again three times. Samples were prepared by diluting recombinant WT and P167S variant C9 to 37.23, 18.62, 9.31, 4.66, 2.33 and 1.165 μg/ml in PBS with or without 200 mm EDTA pH 7.4. 100 μl of samples were added and incubated for 1 h at RT. The plate was washed again three times.100 μl of biotinylated 10E10 (in-house) at 1 μg/ml was added and incubated for 1 h at RT. The plate was washed again three times. One hundred μl of streptavidin–HRP (Abcam Cambridge, UK) diluted 1/10000 in block solution was added and incubated for 1 h at RT. The plate was washed again three times. One hundred μl of TMB solution (9.9 ml phosphate citrate, 3 μl H_2_O_2_, 200 μl TMB, Cambridge, UK) was added and incubated for 5 min at RT. One hundred μl of 10% H_2_SO_4_ was added to each well to stop the reaction. Plates were read at 450 nm.

### Recombinant protein production and purification

DNA coding for human C9 was generated (Invitrogen) and subcloned into the expression vector, pDR2ΔEF1α. DNA was extracted and the P167S polymorphism was introduced using Quikchange II site-directed mutagenesis kit (cat: 200524). The forward primer was CCTGGGCATGGACTCCCTGAGCACCC and the reverse primer was GGGTGCTCAGGGAGTCCATGCCCAGG. Plasmid DNA containing either the WT C9 or P167S mutated C9 sequences was transfected into CHO cells using jetPEI (Polyplus-Transfection SA, Illkirch, France). Stable C9 expressing Chinese hamster ovary (CHO) cell lines were selected.

A C9 affinity purification column was generated on 1 ml HiTrap NHS-activated HP (GE Healthcare, 17-0716-01, Amersham, UK) with the anti-C9 mAb, B7 (Prof. B.P. Morgan, Cardiff University) and according to the manufacturer’s instructions. The column was equilibrated with 20 mm sodium phosphate, 150 mm NaCl pH 7.2 and then loaded with CHO cell supernatant. C9 bound to the column was eluted using 0.1 m glycine pH 2.5 and immediately neutralized using 1 m Tris-HCl pH 8. Proteins were then buffer exchanged into Complement Fixation Diluent (Thermofisher BR16G, Paisley, UK) and concentrated using a Vivaspin 20 (GE Healthcare Life Sciences, Amersham, UK) filter column.

To ensure that the pH elution did not alter the hemolytic activity of affinity purified C9, a comparison of C9-depleted serum reconstituted with affinity purified human C9 versus native serum was undertaken and demonstrated equivalence (not shown).

### SDS-PAGE and western blotting

Samples were diluted appropriately (Fig. 4b, at 100 μ/ml) and run under reducing or non-reducing conditions in commercially produced 10–20% acrylamide Tris-glycine gels (Novex, Fisher scientific, Paisley, UK, 1.0 mm × 10 well). Protein bands were either stained with Coomassie or transferred to nitrocellulose for western blotting. Following transfer, the nitrocellulose was blocked with 5% non-fat milk powder in TBST (Tris-buffered saline 0.1% Tween 20) and then incubated in fresh blocking solution plus polyclonal anti-C9 1:10 000 (Abcam ab71330, Cambridge, UK or CompTech A226, Cambridge, UK). The nitrocellulose was then incubated again, following thorough washing in TBST, with the blocking solution and goat α-rabbit IgG HRP 1:1000 (Abcam, ab6721, Cambridge, UK). After washing again, the nitrocellulose was incubated with SuperSignal West Pico Chemiluminescent Substrate (Thermo Scientific, 34 077, Paisley, UK) and C9 was detected using the Odyssey FC imaging system (LICOR, Cambridge, UK).

### Hemolysis assay

SRBCs (SB068, TCS Biosciences, Buckingham, UK) were prepared and sensitized by adding anti-SRBC stroma (S1389-1VL, Sigma, Gillingham, UK) and then resuspended in GVB (Complement Fixation Test Diluent Tablets (ThermoFisher BR16G, Paisley, UK) and 10% Gelatin (sigma G2500-100 g, Gillingham, UK). Serum, depleted serum and depleted serum reconstituted with C9 were titrated from 20% at 1 in 2 dilutions in GVB on a round-bottomed 96-well plate in triplicate. Each well had 50 μl final volume. Wells for 100% lysis were set up by adding 150 μl of NH_4_Cl red cell lysis solution. A control plate was also set up with a duplicate of each sample dilution prepared in the same buffer plus 50 mm EDTA, to be used as a blank. 50 μl of SRBC (1 × 10^8^ cells/ml in GVB buffer) was added to each well and the mixture incubated at 37°C for 30 min. 100 μl of ice-cold GVB-EDTA buffer was added to stop the reaction, and SRBCs were pelleted by centrifugation at 500*g* for 5 min. Supernatants were transferred to a flat-bottomed 96-well plate, and the absorbance of the supernatants was read at 412 nm. Percent lysis was calculated as 100 × (A412 test-A412 blank)/(A412 100% lysis-A412 blank).

### Reactive lysis assay

Guinea pig erythrocytes (TCS Biosciences, PB031AP, Buckingham, UK) were prepared by suspending 1 ml in 20 ml PBS. They were washed in ice-cold PBS 2× by centrifugation at 500*g* for 5 min at 4°C and then 200 μl of the pellet was diluted in 10 ml ice-cold PBS. 50 μl was pipetted into the wells of a U-bottom 96-well plate on ice. 50 μl/well of PBS was added. 1 μl/well of C5b6 (1 mg/ml, Merck Millipore, 204 906, Gillingham, UK) was added and the plate was incubated at 37°C for 15 min. The plate was put back on ice and 1 μl/well of C7 (at 1 mg/ml) was added. The plate was incubated at 37°C for 15 min and placed back on ice. C8 (1 mg/ml) and C9 (either recombinant WT or a 3:1 WT:P167S mix – 1 mg/ml) were added at the same time 1 μl each per well, diluted in 50 μl PBS. Two wells had PBS added without C8 and C9 to serve as background control and two wells had water added instead of C8 and C9 to represent 100% lysis. The plate was incubated for 30 min at 37°C followed by centrifugation at 1500 rpm for 5 min at 4°C. 100 μl of supernatant was transferred to a flat-bottomed 96-well plate and absorbance was read at 412 nm. Percentage lysis was calculated by: ((Abs sample − Abs background) ÷ (Abs max − Abs background)) × 100%.

### Negative staining EM

MACs assembled on lipid monolayers were imaged using EM, as previously described (42). 10 μl of buffer (120 mm NaCl, 20 mm Hepes pH 7.4) were deposited in a 4 mm diameter well teflon plate and overlaid with 2 μl of lipids in chloroform [DOPC:DOPE 60:40 molar ratio, 1 mg/ml (Avanti Polar Lipids, Alabama, USA)]. The chloroform was evaporated for 1 min and an EM grid (CF400-CU, Agar Scientific, Stansted, UK) was placed carbon-side down onto the solution surface. MAC components were sequentially added to the solution with minimal perturbation as follows: C5b6, C7, C8 [60 nm, 5 min incubation between each new addition, 37°C (Complement Technologies, Texas, USA)] and C9 (1.2 μm, 15 min incubation, 37°C) to achieve a final molar ratio of 1:1:1:20. The grid was then gently peeled off and immediately stained with 2% uranyl acetate. Images were acquired on a Tecnai G2 Spirit electron microscope (Thermo Fisher Scientific, Paisley, UK) with a 2K eagle camera (Thermo Fisher Scientific, Paisley, UK) at ×42 000 magnification (3.7 A/px).

### Polymerization

Purified recombinant C9 (WT and P167S) was subjected to size exclusion chromatography to remove any aggregates and to exchange buffer into assay diluent. On the same day, proteins were removed from ice and incubated at 37°C for 1 h in the presence or absence of EDTA.

### Surface plasmon resonance polymerization

The propensity of recombinant WT and P167S C9 to form poly-C9 was assessed using a Biacore S200 instrument (Cytivia Life Sciences). The anti-human TCC mAb, aE11 (Hycult Biotech, The Netherlands) was captured (94 RU) on flow cell 2 (FC2) of a CM5 Biacore chip using the mouse antibody capture kit (Cytivia; 500RU anti-mouse Ig immobilized). FC1 was activated and blocked as a blank control. Preparations of recombinant WT and P167S C9 were subjected to SEC (Superdex 200, Cytivia Life Sciences) to remove all aggregates and to buffer exchange into Biacore running buffer (10 mm HEPES pH 7.4, 140 mm NaCl, 0.01% surfactant P20). The capture experiment was performed immediately. The temperature of the chip surface was set to 37°C and the protein preparations (300 μg/ml) were flowed across the chip at a very slow flow rate to allow for polymerization (5 μl/min). Capture of TCC on aE11 was monitored; data shown are double-referenced.

### Statistics

Data were analyzed using GraphPad Prism 8.00 for Windows (GraphPad Software, San Diego, CA; www.graphpad.com). Densitometry was carried out using Image studio V5.2 (Licor, UK). For the C9 and sC5b-9 ELISAs, Mann–Whitney tests (Figs 1a, 2a and 3a and c; Supplementary Material, Figs S1 and S3) and Dunn’s multiple comparisons tests (Figs 1b, 2b and 3b) were used to compare medians. A standard unpaired *t*-test was used to compare recombinant expression of C9 in CHO cells (Fig. 4). A Wilcoxon matched-pairs test was performed to compare lytic activity (Figs 6 and 7).

## Supplementary Material

McMahon_et_al_supplementary_submission_ddab086
