# Supplementary material for: The rare C9 P167S risk variant for age-related macular degeneration increases polymerization of the terminal component of the complement cascade
Source: Hum Mol Genet. 2021 Mar 30;30(13):1188–99. doi: 10.1093/hmg/ddab086 (PMC8212764; doi:10.1093/hmg/ddab086)
Supplement: McMahon_et_al_supplementary_submission_ddab086 [file mcmahon_et_al_supplementary_submission_ddab086.pdf]

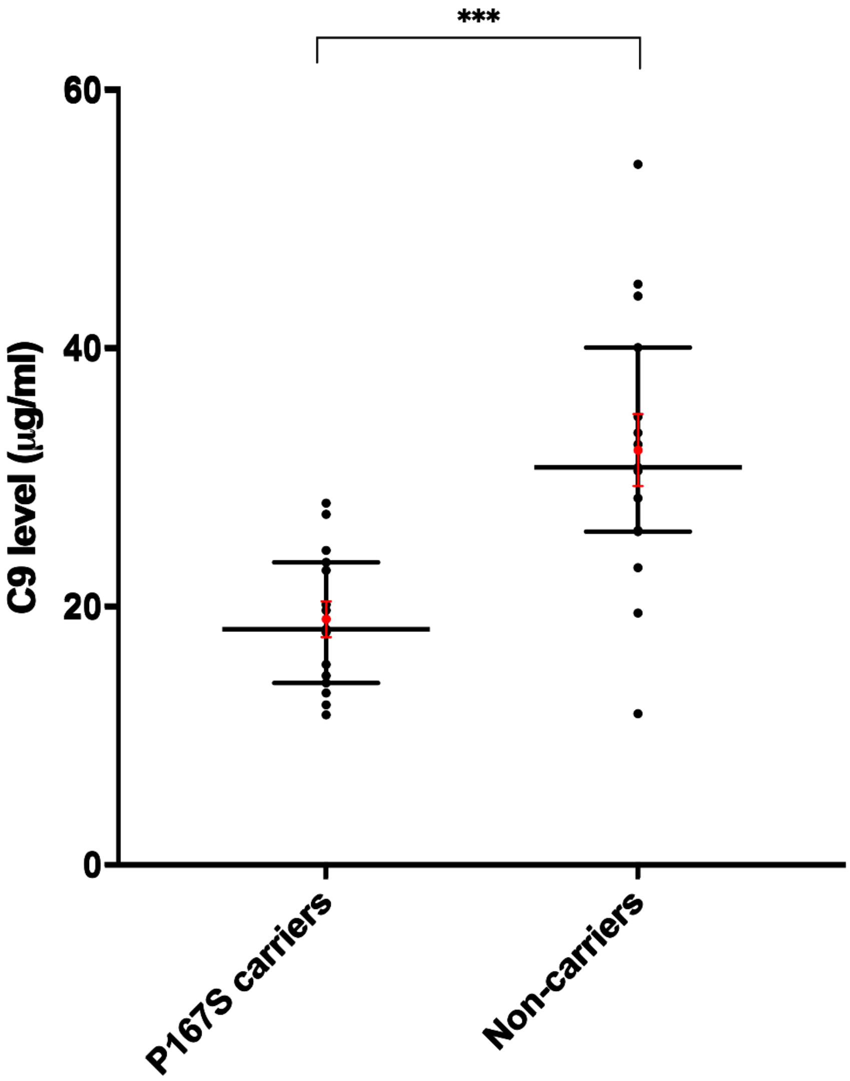

Supplementary Figure 1: Plasma C9 levels using the 10E10 C9 ELISA, grouped according to P167S status. 30 samples were randomly selected. C9 plasma levels measured using an in-house 10E10 ELISA. Statistics shown include the mean with SEM (red), interquartile range and median (black bars). The results show that overall those with the P167S variant had significantly lower median levels of C9 than those without the variant (18.3µg/ml vs 30.8µg/mL, P=0.0003). Statistically significant results are indicated by (***). ***P<0.001.

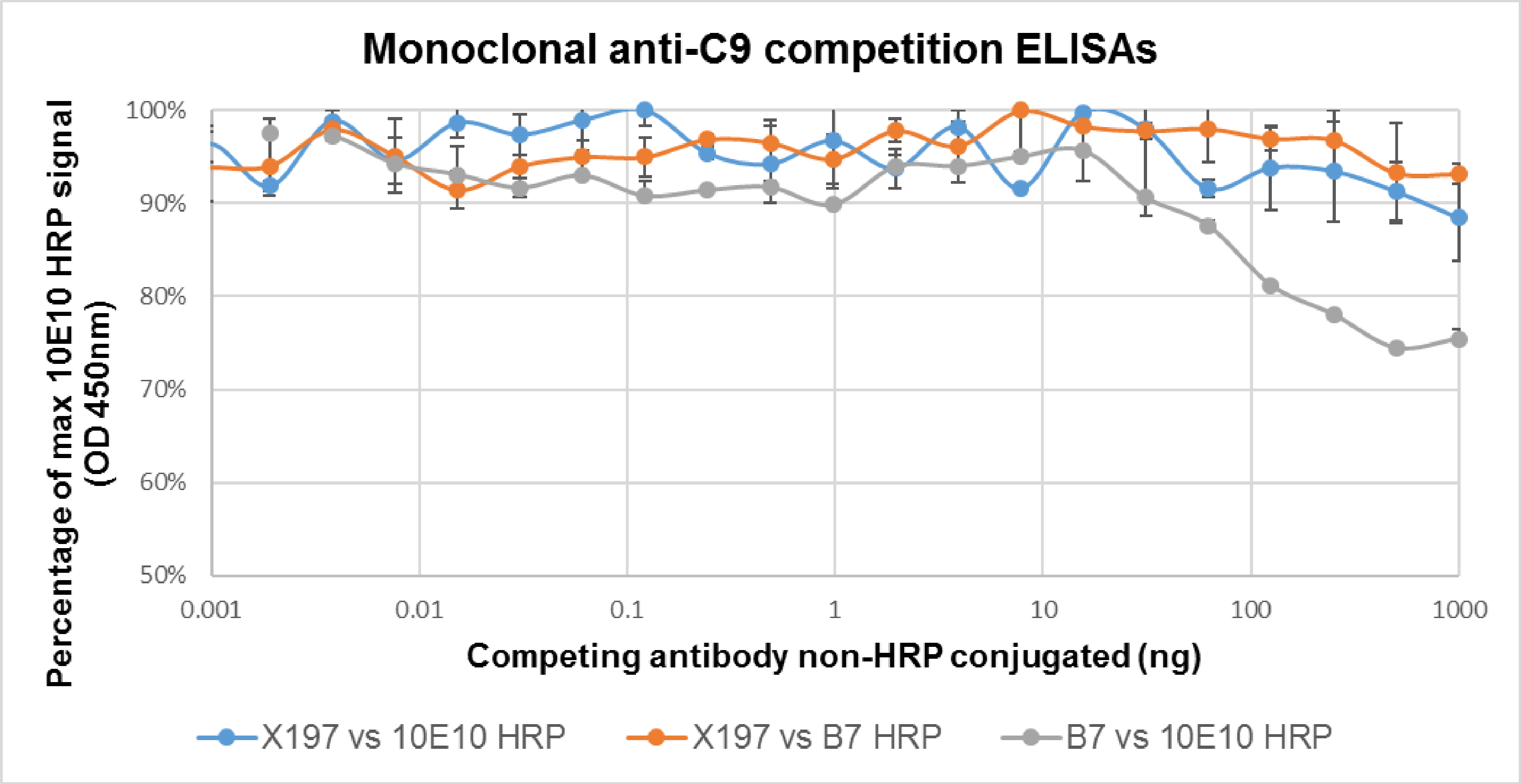

Supplementary Figure 2: Competition between B7, 10E10 and X197 anti-C9 monoclonal antibodies. C9 was coated onto the plate at 1µg/ml and the competing antibody added at a range of concentrations from 1000ng to 0. The HRP-conjugated monoclonal anti-C9 antibody was then added, B7 HRP or 10E10 HRP. If there was no competition between the added competing monoclonal anti-C9 and the HRP-conjugated monoclonal anti-C9 then signal detected would be unaffected and remain at 100%. When B7 and 10E10 mAb were in competition, there was a loss of signal suggesting overlapping epitope targets. There was no competition between X197 and 10E10 or between X197 and B7.

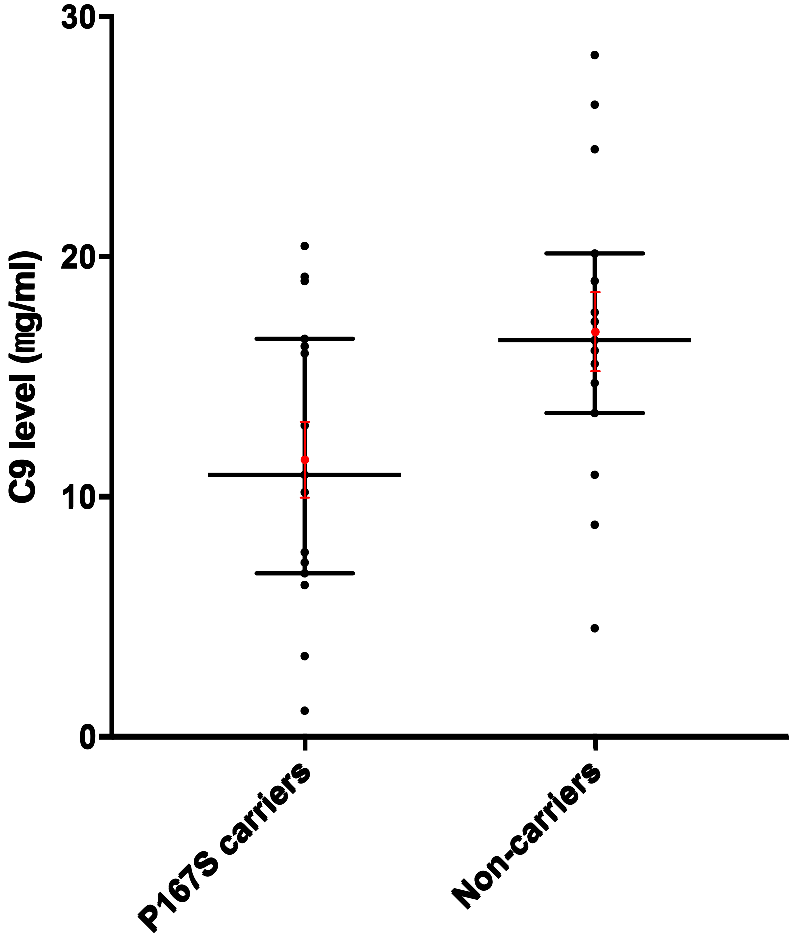

Supplementary Figure 3: Plasma C9 levels using the X197 C9 ELISA, grouped according to P167S status. 30 samples were randomly selected. C9 plasma levels measured using ELISA antibodies and method replicated from Kremlitzka et al. Statistics shown include the mean with SEM (red), interquartile range and median (black bars). The results show that overall, those with the P167S variant had lower median levels of C9 than those without the variant (10.9µg/mL vs 16.9µg/mL, P=0.054).

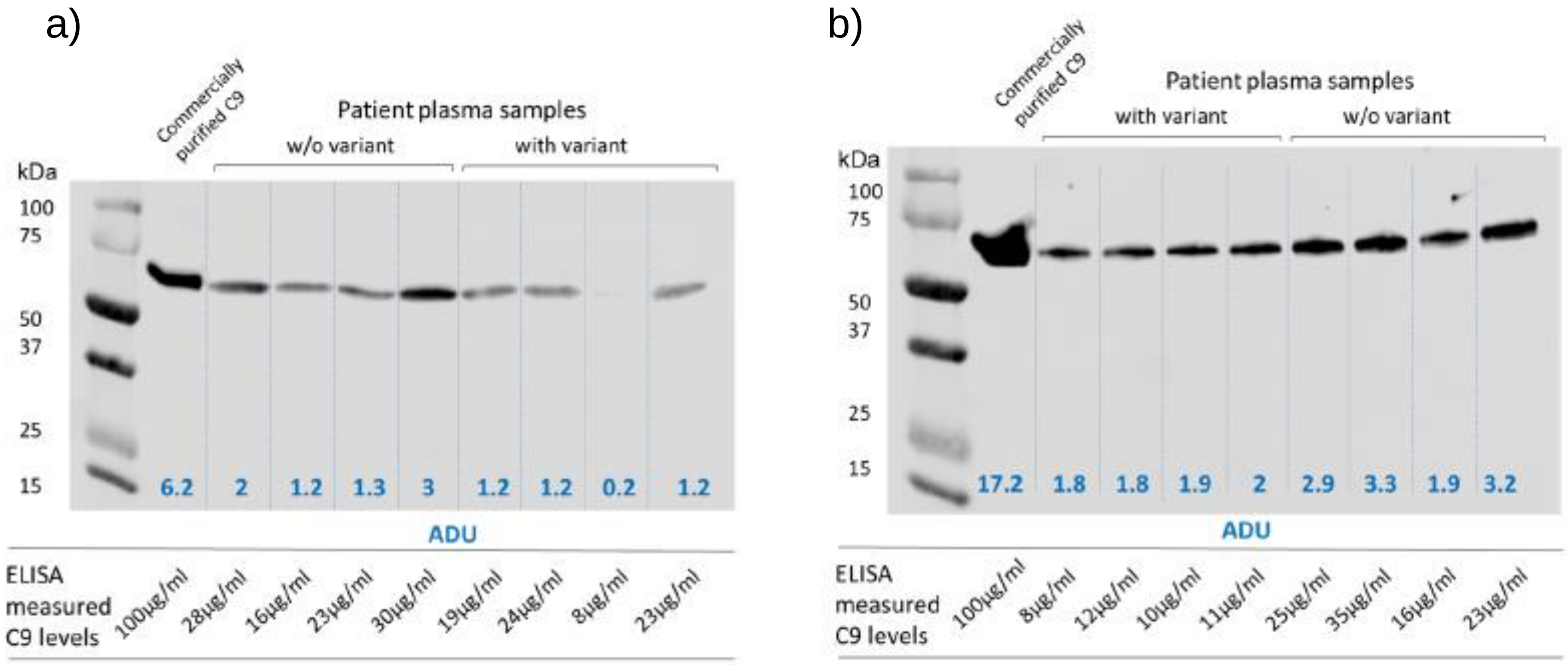

Supplementary Figure 4: ***Densitometry analysis of western blots of C9 levels in plasma samples*** *16 plasma samples from the ELISA-analysed cohort were randomly selected according to variant status and analysed using a western blot technique and imaged using a Licor Odyssey. The Licor calculated arbitrary density units (ADU) to compare the signal detected from each sample (blue). The higher the ADU, the higher the fluorescence of the sample and the greater the C9 content. The levels measured previously by the B7 ELISA are shown below the blots. (a) samples were diluted 1/100 and detected using Ab71300 rabbit poly anti-C9 (Abcam). (b) samples were diluted 1/ 1000 and detected using A226 goat poly anti-C9 (CompTech).*

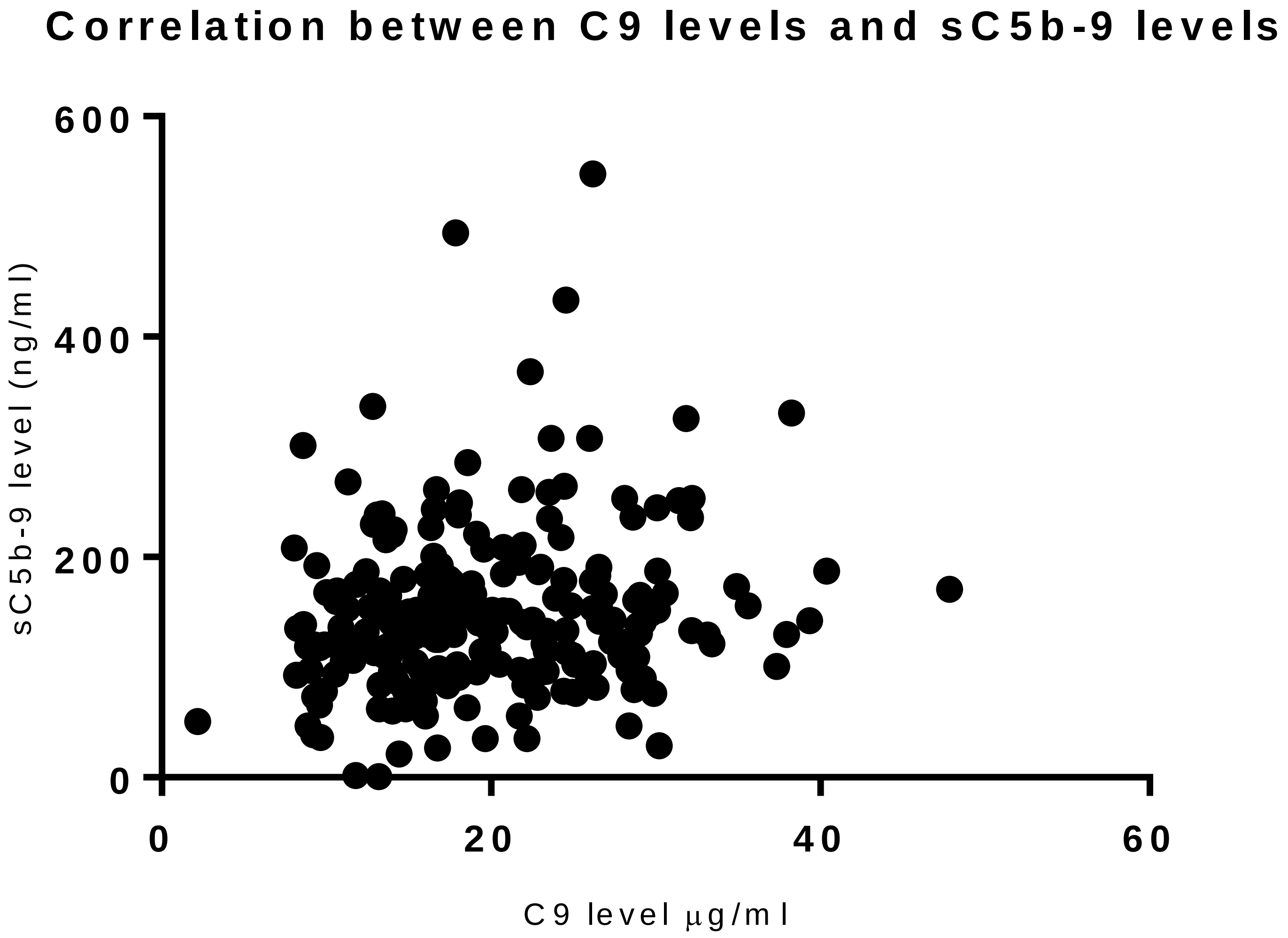

Supplementary Figure 5: Correlation between C9 levels and sC5b-9 levels

Pearson's rank correlation = 0.175 between C9 levels and sC5b-9/TCC levels in samples with a significance of P=0.0079.

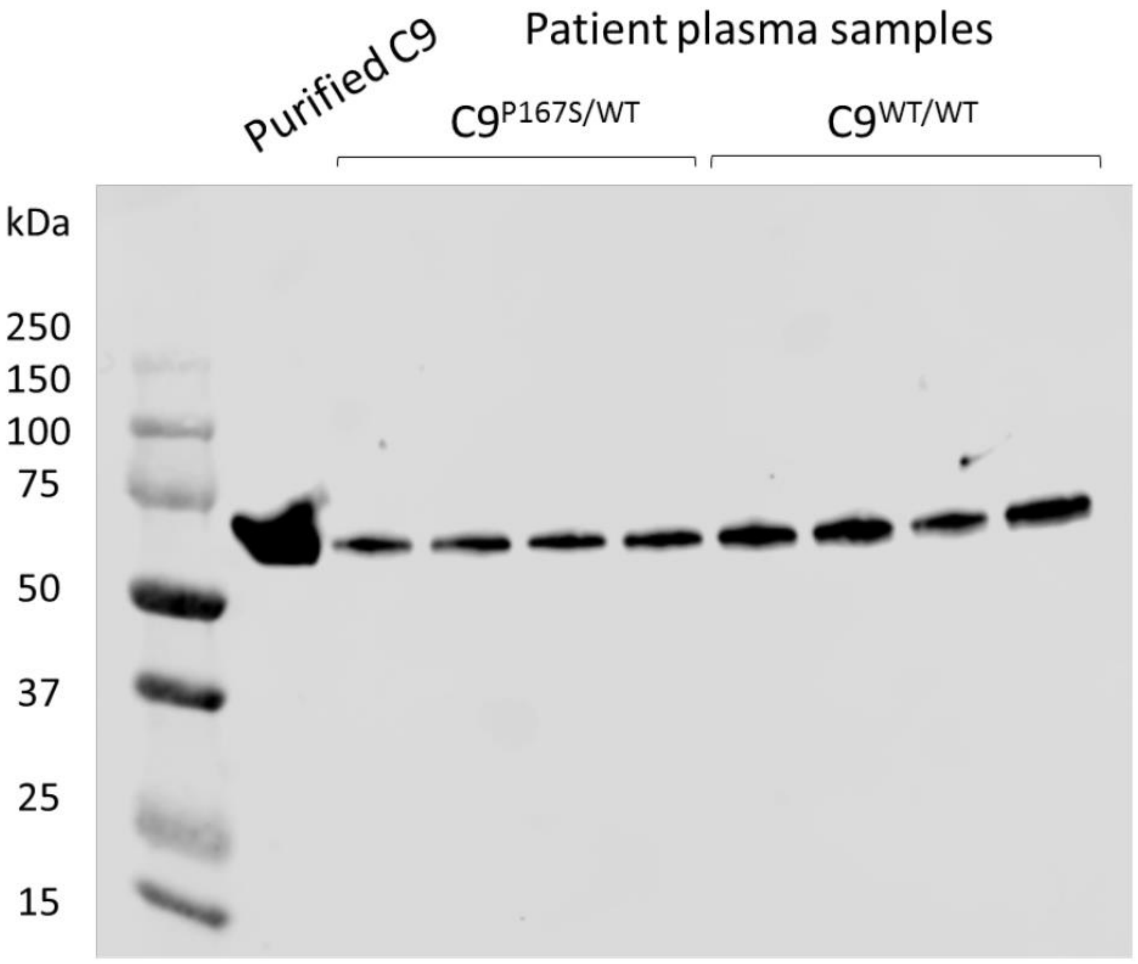

Supplementary Figure 6: Plasma samples from P167S variant carriers and non-carriers show no polymerised/aggregated C9. Western blot of four plasma samples from P167S variant carriers (C9P167S/WT) and four from non-carriers (C9WT/WT) diluted 1/100. Detected using rabbit polyclonal anti-C9 (Abcam, ab71330) and HRP-conjugated goat anti-rabbit Ig (Abcam, Ab6721). Visualised using Licor Odyssey.
